# Where should we offer mass drug administration for trachoma?

**DOI:** 10.5694/mja2.51752

**Published:** 2022-10-26

**Authors:** Jaki Adams, Sung Hye Kim, Anthony W Solomon

**Affiliations:** 1 The Fred Hollows Foundation, Darwin, NT; 2 World Health Organization Regional Office for the Western Pacific, Manila, Philippines; 3 Hanyang University, Seoul, Republic of Korea; 4 World Health Organization, Geneva, Switzerland

Elimination programs should be guided by the prevalence of markers of infection, not of disease

Trachomatous trichiasis can be devastating: it deforms the eyelid, scars the cornea, and blinds the eye.^1^ It disables the individual and impoverishes their family; quality of life is severely impaired.^2^ As restoring sight to a dry eye with a vascularised cornea using keratoplasty is difficult, these effects are generally irreversible. Most people blinded by trachomatous trichiasis live in poor, remote communities without the visual rehabilitation and support services available in major cities.

The cause of all this suffering is the bacterium *Chlamydia trachomatis*. Ocular *C. trachomatis* strains are passed from eye to eye in overcrowded bedrooms in communities where access to water, sanitation, and hygiene is inadequate.^3^ About 150 ocular *C. trachomatis* infections are required to precipitate trichiasis.^4^ Given that most infections are in children, mostly of pre-school age,^5^ children blinded later in life by trachoma must be very frequently infected.

To determine which communities need interventions, including mass drug administration of anti-chlamydial antibiotics, to reduce the risk of future trachomatous blindness, we would ideally measure the incidence of infection. But most infections in children are subclinical, being at most mildly symptomatic.^3^ Alternatively, we could measure infection prevalence in population-based surveys, but this approach has also been problematic; the sensitivity of tests other than those employing polymerase chain reaction (PCR) or similar nucleic acid amplification technologies is inadequate, and PCR testing has been unavailable or unaffordable for programs serving trachoma-endemic communities in most countries.^6^ Instead, programs are guided by the prevalence in children of the active (inflammatory) trachoma sign, trachomatous inflammation—follicular (TF).

Circumstances, however, are changing. First, the number of people living in trachoma-endemic areas has shrunk, from an estimated 1517 million in 2002 to 125 million in June 2022.^7^ Fewer than 20 000 Australians live in trachoma-endemic areas,^7^ almost all in remote Aboriginal and Torres Strait Islander communities.^8^ Second, PCR testing has become more widely available. Third, we now know that as the prevalence of TF falls, so too does its specificity for conjunctival *C. trachomatis* infection.^9^ Fourth, we are learning that TF prevalence is particularly likely to overestimate the burden of *C. trachomatis* in parts of the Pacific.^10^ Fifth, although the prevalence of macrolide-resistant bystander organisms declines after discontinuing periodic azithromycin mass drug administration for trachoma elimination purposes,^11^ unnecessary use of antibiotics should always be discouraged.

The report by Lynch and colleagues in this issue of the Journal^12^ is consequently a welcome addition to the literature. In a Queensland community categorised (on the basis of TF prevalence in children) as qualifying for antibiotic mass drug administration, only one of 28 cases of TF identified in 1–9-year-old children across three annual rounds of examination was PCR-positive for *C. trachomatis. Haemophilus influenzae* and *Staphylococcus aureus* were more frequently identified (by conventional culture). The prevalence of circulating anti-*C. trachomatis* antibodies in children and of easily visible conjunctival scarring in teenagers (a forerunner of later trachomatous trichiasis) were each low.^12^

Is this community still trachoma-endemic? Yes: it still includes adults with trachomatous trichiasis, who should be offered a simple corrective operation to reduce the risk of corneal opacification. But is antibiotic mass drug administration indicated for trachoma elimination purposes? We agree with Lynch and colleagues that it is not.

Interpreting infection and antibody data for trachoma programs is inherently complex. Someone with a urogenital *C. trachomatis* infection can transfer the organism to their own eyes or those of their children or other contacts on unwashed hands. Such infections can elicit phenotypic and serological responses identical to those of ocular *C. trachomatis* infections of the conjunctivae, but are not associated with endemic trachoma and trachomatous trichiasis.^3^ We do not know whether the PCR and anti-*C. trachomatis* antibody signals detected by Lynch and colleagues were responses to ocular or urogenital *C. trachomatis*. Sequencing of eye swabs could help resolve this question, but is not required for deciding whether antibiotic mass drug administration is warranted.

Like the national guidelines in Australia, international guidance for antibiotic use in trachoma programs is based on TF prevalence in children, as is one of the World Health Organization criteria for trachoma elimination.^13^ Vanuatu has recently become the fourteenth country to eliminate trachoma as a public health problem;^14^ its case for validation relied in part on the TF found in children’s eyelids not being associated with PCR evidence of *C. trachomatis* infection, high levels of anti-*C. trachomatis* antibodies in children, or much conjunctival scarring in adolescents.^15,16^ As Lynch and colleagues advise for Australia, international guidance for trachoma programs should be re-assessed.
